# Effect of Preliminary Treatment by Pulsed Electric Fields and Blanching on the Quality of Fried Sweet Potato Chips

**DOI:** 10.3390/foods12112147

**Published:** 2023-05-26

**Authors:** Caiyun Liu, Minming Lv, Huihui Du, Haoyu Deng, Lu Zhou, Piaoran Li, Xuxian Li, Baoguo Li

**Affiliations:** School of Health Science and Engineering, University of Shanghai for Science and Technology, Shanghai 200093, China; jnyzlcy@126.com (C.L.);

**Keywords:** frying, blanching, pulse electric field, sweet potato chips

## Abstract

The effects of pulsed electric fields (PEF) and blanching pretreatments on frying kinetics, oil content, color, texture, acrylamide (AA) content, and microstructure have been investigated in this paper. The total PEF pretreatment duration was t_PEF_ = 0.2 s with an intensity of *E* = 1 kV/cm; blanching was studied at 85 °C for 5 min. The results demonstrated that pretreatment significantly reduced the moisture ratio and oil content by 25% and 40.33%, respectively. The total color change Δ*E* value of the pretreated samples was lower than that of the untreated samples. In addition, pretreatment increased the hardness of the sample after frying, and the AA content in the fried samples pretreated with PEF + blanching was reduced by approximately 46.10% (638 μg/kg). Finally, fried sweet potato chips obtained by the combined pretreatment exhibited a smoother and flatter cross-sectional microstructure.

## 1. Introduction

Fried foods are significantly popular worldwide due to their unique flavors. Sweet potato is a naturally nourishing food rich in protein, fat, polysaccharides, phosphorus, calcium, potassium, carotene, vitamin A, vitamin C, vitamin E, vitamin B_1_, vitamin B_2_, and eight amino acids. Sweet potatoes are widely cooked by deep-frying and consumed as French fries and chips [1,2]. Studies have shown that a high fat and calorie intake may lead to metabolic disorders, resulting in an increased risk of hypertension, cardiovascular disease, diabetes, and cancer [3,4,5]. Therefore, there is currently a strong demand for high-quality fried foods that are likely to reduce the oil intake and formation of carcinogen acrylamide content.

Recommended mitigation measures, such as magnetic fields, microwaves, and UV-C, have been used to control the acrylamide content in potatoes and potato semi-finished products. Sobol et al. found that potato tubers exposed to UV-C radiation caused an increase in the acrylamide content; however, soaking the semi-product in water resulted in a decrease in the acrylamide content in French fries [6]. Polysaccharides (alginate, pectin, and chitosan) are used in food frying processes and can inhibit the formation of acrylamide by up to 54%, 51%, and 41%, respectively [7]. Conventional blanching pretreatment is widely used to improve the quality of fried sweet potato chips [8]. During blanching, gelatinization of starch can reduce oil intake during the frying of potato chips [9]. In addition, the sugar and asparagine contents of potatoes can be significantly reduced by hot blanching pretreatment, thus reducing the formation of acrylamide [9,10]. However, heat treatment involves high energy consumption and may lead to unexpected quality changes, such as the loss of soluble nutrients and nutrient deactivation (polyphenols) [11,12]. Pulsed electric field (PEF) is a novel non-thermal physical treatment technique that discharges a sample between two electrode plates by applying a high-voltage pulse, mainly utilizing an electroporation mechanism [13,14,15]. PEF pretreatment induces the formation of transient microspores in the lipid bilayer of the cell membrane, which improves cell permeability and forms more channels for water to migrate outside, accelerating the diffusion of water and reducing oil absorption [15,16]. Moreover, PEF can soften the tissue and smoothen the surface, further reducing the oil absorbed during frying [17]. Ostermeier et al. found that PEF allows better removal of water, decreases color change, and reduces the acrylamide content of potato chips simultaneously [18]. More than 50% of glucose and 48% of asparagine can be reportedly removed from potato chips after PEF pretreatment, resulting in lower levels of acrylamide [19]. Zhang et al. [20] found that PEF pretreatment improved the color of fried potato chips and reduced their acrylamide content to 1533.75 ng/g. However, the combined effects of PEF and blanching treatment on the physicochemical properties of fried sweet potato chips remain unclear.

This study aimed to evaluate the preliminary effects of PEF, blanching, and their combinations on deep-fried sweet potato chips. The effect of pretreatment on the cellular microstructure was studied using scanning electron microscopy (SEM). The quality of the fried sweet potato chips was investigated and compared with that of untreated samples. Finally, the influence of different pretreatments on the acrylamide content of sweet potato chips was determined to inhibit acrylamide formation during deep frying.

## 2. Materials and Methods

### 2.1. Materials

The sweet potatoes (Liu Ao Red Sweet Potato) were purchased from a local market in Shanghai, China and were stored in a refrigerator at 4 °C. All experimental data were collected within one week of purchase. Fresh sweet potatoes were cleaned, sliced (27 mm in diameter and 3 mm in thickness), and sampled using a stainless steel circular mold. The initial moisture content of sweet potatoes (*W_i_* = 3.67 ± 0.1 db or 0.785 ± 0.1 wb) was determined by drying the samples at 105 °C in the oven (DHG-9245A, HuiTai, Shanghai, China).

### 2.2. PEF Pretreatment

**Figure 1:**
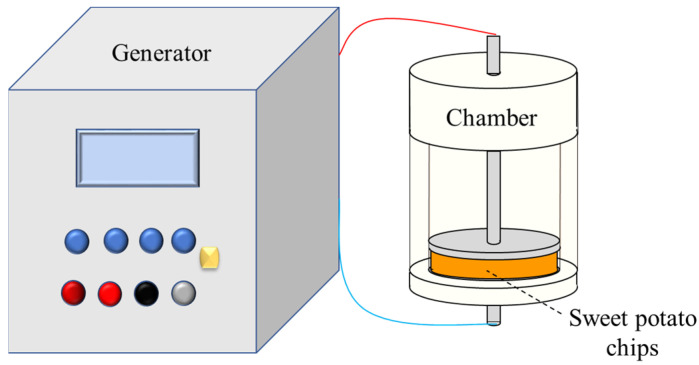
PEF treatment procedures applied to the sweet potato tissue.

A PEF generator delivering monopolar pulses (1500 V-1A, Service Electronique USST, Shanghai, China) was used. Figure 1 presents the PEF-applied treatment procedures for sweet potato slices. The processing chamber (Teflon cylindrical tube, industrial processes workshop, USST, Shanghai, China) consisted of two parallel stainless steel electrodes and had a diameter of 41.5 mm and a depth of 100 mm. The electric field intensity was *E* = 1k V/cm and there were a series of *N* = 200 trains. Each train consisted of *n* (=50) pulses with a pulse width of *t_i_* = 20 μs and a frequency of 10 Hz. The total time of the PEF treatment was calculated as *t_PEF_* = *N·n·t_i_* = 0.2 s. The applied protocol allowed for obtaining a high level of electroporation of sweet potato tissue based on our preliminary studies. The temperature elevation inside the samples never exceeded 5 °C. The energy input of the PEF pretreatment was 9.47 ± 0.5 kJ/kg, calculated as follows [21]:

(1) $$E_{e}=U\cdot I\cdot \frac{t_{PEF}}{1000M}$$

where, *U* is the voltage (V), *I* is the flowing current (A) obtained from the display screen of the generator, *t_PEF_* is the total duration of the PEF treatment (s), and *M* (kg) is the mass of the sample.

### 2.3. Blanching Pretreatment

Sweet potato slices were heated and stirred at 85 °C for 5 min on a ceramic heating plate HJ-2A (Guohua, Changzhou, China) by following the method of Timolsina et al. [22], with slight modifications. After the blanching treatment, the surface water was removed, and the potato slices were cooled to an ambient temperature for frying.

### 2.4. Frying

Different pretreated and untreated sweet potato slices were then fried in hot sunflower oil contained in an electro-thermal blast furnace at 150 °C (HY-81, Foshan Nanhai Gangyang Electromechanical Equipment Co., Ltd., Foshan, China) with a sample/oil mass ratio of 1/60 for 6 min. Previous results showed that when frying at 150 °C, the PEF pretreatment could significantly decrease the acrylamide content by 70% of potato chips [23]. The mass (*m*) of the samples was periodically controlled during frying. The moisture ratio (*MR*) of a sample during frying was calculated as follows:

(2) $$MR=\frac{m_{t}}{m_{i}}$$

where, *m_i_* is the initial moisture content and *m_t_* is the moisture content after frying that was obtained using a moisture analyzer (HC103, Mettler Toledo Instruments Co., Ltd., Shanghai, China).

### 2.5. Analysis of the Samples

#### 2.5.1. Oil Content

The oil content of the fried sweet potatoes chips was measured using a low-field nuclear magnetic resonance (LF-NMR) spectrometer (PQ001-020-015V, Niumag Corporation, Suzhou, China), with a frequency field of 20 MHz and a temperature of 32 ± 0.01 °C. For these measurements, the nuclear magnetic field strength was 0.5 ± 0.08 T. It was measured by placing the sample in a 15 mm glass tube and inserting it into the NMR probe. The Carr-Purcell-Meiboom-Gill (CPMG) pulse sequence was applied to measure the transverse relaxation time (*T*_2_). Typical pulse parameters included a sampling frequency of 250 kHz, repetition time of 2000 ms, echo count of 5000, echo time of 1 ms, and repeat scan times of 4 [24]. Standard curves were constructed as follows.

**Figure 2:**
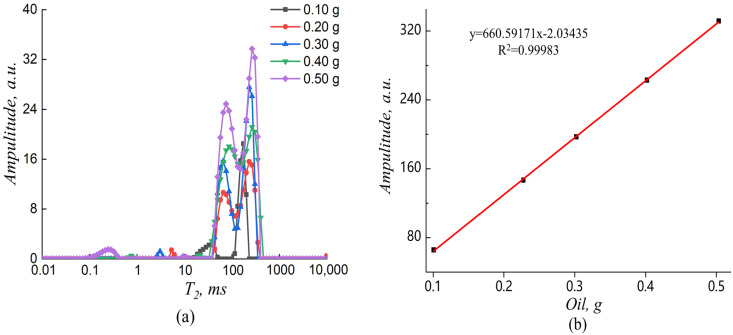
Distribution of the transverse relaxation time (*T*_2_) spectra of the different masses of sunflower oil (**a**), and standard curve of oil (**b**). *R*^2^ is the coefficient of determination; the equation presented in the chart indicates the trend line.

The sunflower oil was weighed in 15 mm glass tubes with weights of 0.1, 0.2, 0.3, 0.4, and 0.5 g, which were measured in a water bath at 32 °C for 5 min to obtain the amplitude corresponding to the different masses of oil. Figure 2a presents the distribution of the transverse relaxation time (*T*_2_) spectra for different masses of sunflower oil. The relaxation signal shown in the figure can be entirely attributed to the protons in the oil molecule and is composed of a small and large characteristic peak. The oil peak emerged in the range of 12–464 ms, providing a basis for distinguishing the proton signals of water and oil in the sample. The signal amplitude increased as the oil mass increased. The linear equation fitted to the mass and amplitude of the oil was y = 660.59172x − 2.03436, with *R*^2^ = 0.99983, indicating that the mass of the oil linearly and sufficiently correlated with the peak area (Figure 2b). The intensity of the peak signal is linearly related to the mass of oil. Therefore, the correlation between the peak intensity and mass of oil can be determined by calibrating the sample to obtain the oil content (*O_f_*) of the sample.

#### 2.5.2. Color

The color of the samples was determined using a colorimeter (CR-400; Konica Minolta Investment Co., Ltd., Shanghai, China). The color parameter coordinates L* (whiteness or brightness), a* (redness or greenness), and b* (yellowness or blueness) were used to describe the color of the samples [25]. Hunter values (L*, a*, b*) were monitored on the surfaces of untreated and pretreated fresh and fried samples. The total color difference Δ*E* was used to express the overall color change during the thermal process and was calculated by using Equation (2) as follows:

(3) $$∆E=\sqrt{{\left(L^{*}-{L_{0}}^{*}\right)}^{2}+{\left(a^{*}-{a_{0}}^{*}\right)}^{2}+{\left(b^{*}-{b_{0}}^{*}\right)}^{2}}$$

where, ${L_{0}}^{*}$, ${a_{0}}^{*}$, and ${b_{0}}^{*}$ indicate the color parameters of the fresh samples;$L^{*}$, $a^{*}$, and $b^{*}$ indicate the color parameters of the fried samples.

#### 2.5.3. Texture

To obtain the hardness of the chips, a texture analyzer (TA-XT PlusC, Stable Micro Systems Co. Ltd., Manchester, UK) with the texture profile analysis (TPA) mode was used [23]. The sample was placed just below the probe and tested with a P/0.25 S spherical probe while maintaining the sample placed in the same direction for each test. The parameters were set as follows: pre-test speed of 1.0 mm/s, mid-test speed of 0.5 mm/s, post-test speed of 10.0 mm/s, distance of 1.5 mm, and trigger force of 5 g. The average force between the first peak and 1 s was expressed as the hardness of the potato chips. The textural parameters of hardness were calculated from the TPA curve using the Texture Exponent software (Stable Micro Systems Co. Ltd., Manchester, UK).

#### 2.5.4. Liquid Chromatography–Tandem Mass Spectrometry/Mass Spectrometry Analysis of Acrylamide

The acrylamide determination of the fried sweet potatoes was performed as described by Liu et al. [23], with slight modifications. An LC-MS/MS system (Agilent7890, Santa Clara, CA, USA) equipped with an auto-sampler and Atlantis C_18_ columns (5 μm, 2.1 mm I.D. × 150 mm) was used; 50 g of the fried sample was obtained, pulverized by a food processor (Elfin2.0, Shengzheng, China) and stored frozen at −20 °C. A total of 10 μL of a 10 mg/L^13^C_3_-acrylamide internal standard working solution and 10 mL of ultrapure water were added to 2 g of pulverized samples, shaken for 30 min, and then centrifuged at 4000 r/m for 10 min using a centrifuge (Medifuge™, Carlsbad, CA, USA); the supernatant was then collected. A matrix solid-phase dispersion extraction method was used for the purification. The elution was in isocratic mode using a mixture of 0.1% *v*/*v* formic acid and methanol (99.5/0.5, *v*/*v*) as the mobile phase at a flow rate of 2 mL/min; the injection volume of the sample was 25 μL. A standard series of working solutions was injected into the LC-MS/MS system and the peak areas of the corresponding acrylamide, and its internal standard were measured. The results of the fried sweet potatoes were expressed in μg/kg.

#### 2.5.5. Scanning Electron Microscope

The microstructure of the sample was obtained using an SEM instrument (Thermo Scientific Apreo 2C, Waltham, MA, USA) equipped with a low-Vac mode, an accelerating voltage of 10 kV, and an amplification of 500. Ten images from three different samples were analyzed for each experiment.

### 2.6. Statistical Analysis

Data were obtained from five replicates. Results are presented as the mean ± standard deviation. One-way analysis of variance (ANOVA) was used to analyze the effect of pretreatment using the IBM SPSS Statistics 26 analysis software (IBM Institute, New York, NY, USA). All statistical analyses were performed with a significance level of 0.05 using Duncan’s multiple range tests. A software package, Table Curve 2D, version 5.01 (Systat Software, San Jose, CA, USA), was used to fit the curve to obtain the relevant correlation coefficients (*R*^2^) and parameters.

## 3. Results and Discussion

### 3.1. Effect of Pretreatment on the Moisture Ratio of Sample

**Figure 3:**
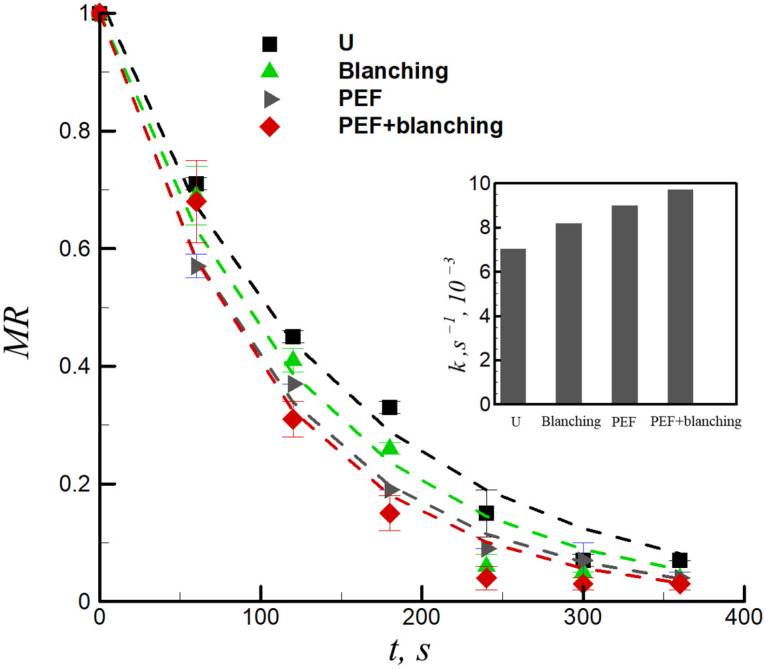
Moisture ratio (*MR*) versus the frying time for untreated (U), blanching-pretreated, PEF-pretreated, and combination of PEF + blanching-pretreated samples. Inset demonstrates the frying rate constant (*k*) for the untreated and pretreated samples.

Figure 3 presents the relationship between the moisture ratios and frying time of sweet potato chips with untreated, blanching-pretreated, PEF-pretreated, and a combination of PEF + blanching-pretreated samples during frying (0–6 min). The moisture ratios of the sweet potato slices was apparently significantly affected by the various pretreatment methods. After frying for 6 min, the moisture ratios of untreated, blanching-pretreated, PEF-pretreated, and combination of PEF + blanching-pretreated samples were 0.07, 0.04, 0.04, and 0.03, respectively. This is consistent with the results of Zhang et al. [20], who evaluated the effects of blanching pretreatment and PEF on the physicochemical properties of French fries. The PEF and blanching pretreatments affect the cell integrity and permeability, which directly leads to differences in the moisture ratio after frying. The development of the moisture ratio with the frying time was fitted using the empirical Henderson and Pabis equation (Equation (4)) (Figure 3, dashed lines). The *R*^2^ values of the untreated and pretreated samples were relatively high (*R*^2^ = 0.980 − 0.997). The values of the frying rate constant *k* as a function of pretreatment ranged from 7.03 × 10^−3^ s^−1^ to 9.71 × 10^−3^ s^−1^ (inset of Figure 3). The results demonstrated that the combination of PEF + blanching pretreatments caused a significant increase in the frying rate constant (*p* < 0.05). The cell membrane electroporated by PEF can promote water migration from the core to the surface, which also increases the mass transfer during the frying process, thereby increasing the frying rate constant [26]. Similarly, blanching disrupts the plant cell walls by degrading pectin, thereby increasing cell permeability [27]. Compared to the untreated samples, the combination of PEF + blanching pretreatment increased the frying rate of the samples by 38.12% (inset of Figure 3), which demonstrates that they have a synergistic effect on water evaporation during frying.

(4) MR=−Aexp(−kt)

where, *k* is the frying rate constant, and s^−1^ and *A* are the frying coefficients.

### 3.2. Effect of Pretreatment on Oil Content of the Sample

**Figure 4:**
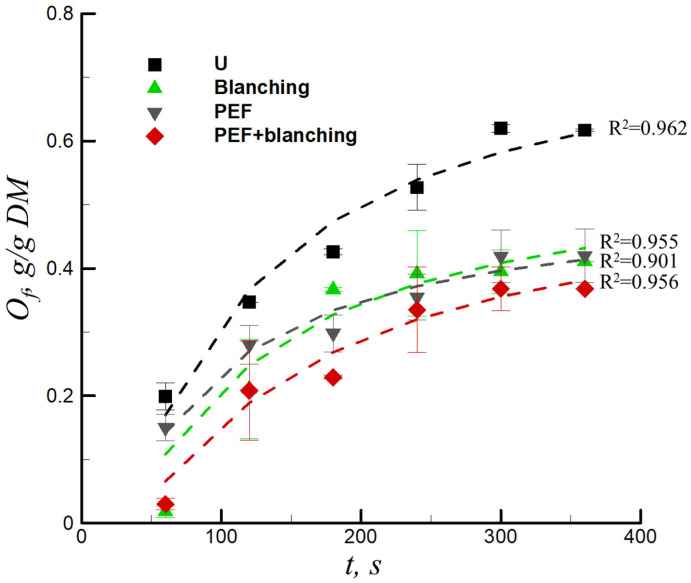
Oil content versus frying time for untreated (U), blanching-pretreated, PEF-pretreated, and combination of PEF + blanching-pretreated samples, *DM* is dry metter.

Deep-frying is a mass- and heat-transfer process that involves water evaporation and oil absorption [28]. Figure 4 demonstrates the development of the oil content (*O_f_*) for fried sweet potatoes that are untreated, blanched, PEF, and PEF + blanched pretreated samples; the dashed lines were obtained by fitting the data with Equation (5). The relevant correlation coefficients (*R*^2^) were all above 0.902, and the parameters of the equation fit for the untreated, blanched, PEF, and PEF + blanched pretreated samples are presented in Table 1. In all cases, the oil content increased as the frying time increased. Compared to the untreated chips, the oil content of the sweet potato chips significantly decreased by 33.38%, 31.90%, and 40.33% with blanching, PEF, and the combination of PEF + blanching pretreatments, respectively. This can be explained by the higher frying rate with PEF pretreatment (Figure 3), which forms a crust on the surface of the sweet potato chips, thereby reducing the oil absorption during frying [15,17,29]. PEF may also cause more cytoplasm to flow out of the cell, forming a water vapor barrier layer on the surface and ultimately reducing oil absorption [30]. In addition, the smoother tissue surface of the samples resulting from the PEF treatment may lead to less oil adhesion after frying due to oil content reduction [31,32]. Similarly, starch gelatinization occurred during the blanching pretreatment, which prevents more oil penetration during the frying process compared to the untreated samples [8,16]. Zhang et al. [26] found that the combination of PEF + blanching pretreatment decreased the oil content of the French fries by 13.8%. Liu et al. [2] investigated the physical–chemical properties of fried sweet potato tubers with PEF pretreatment and found that the oil content decreased by 18.3% with a PEF pretreatment at 1.2 kV/cm and frying temperature of 190 °C.

(5) $${lnO}_{f}=a+\frac{b}{t}$$

where, *a* and *b* indicate the constants of the models.

### 3.3. Effect of Pretreatment on the Total Color Change of Sample

**Figure 5:**
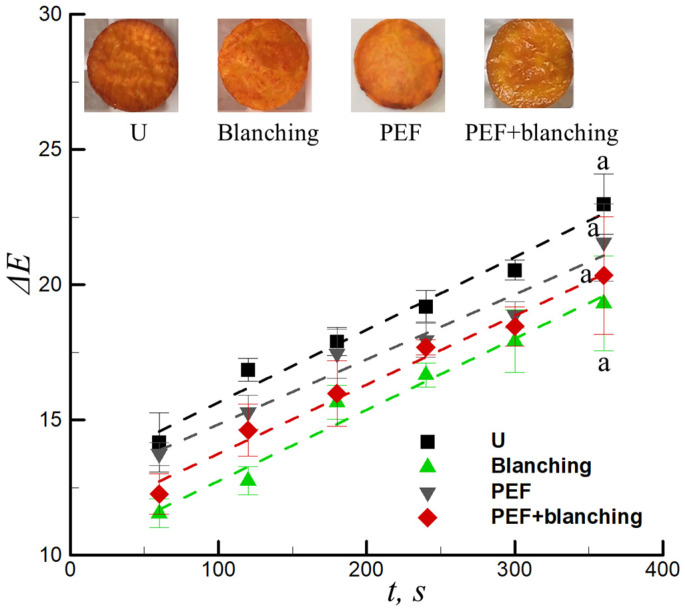
Total color change versus the frying time for untreated (U), blanching-pretreated, PEF-pretreated, and combination of PEF + blanching-pretreated samples. (inset indicates untreated and pretreated samples). ^a^ Values with same superscript letters means no significantly difference.

Color is the basic characteristic used to evaluate the quality and acceptance of fried food, which affects the consumers’ choice of products [33]. Choi et al. demonstrated that Δ*E* > 2 indicates that the color of the sample changed compared to the raw material [34]. The tendency curves of the total color change with frying time were fitted using the linear equation in Equation (6) (dashed lines) (Figure 5). The relevant correlation coefficients were *R*^2^ ≥0.96 (Table 2). The linear equation appears to precisely describe the obtained data for the total color change value. The color of the samples gradually changed from orange to brown during frying. The apparent change (Δ*E*) was indicated by values increasing from 14.16 to 22.98 within a frying time of 1–6 min for the untreated samples (Figure 5). After frying, the total color change Δ*E* of the blanched, PEF, and PEF + blanched pretreated samples were 19.26, 21.57, and 20.34, respectively (Figure 5). The color change in the samples was mainly due to the occurrence of the Maillard reaction during frying. Moreover, the degree of browning depends on the amount of reducing sugars and amino acids on the surface of the samples [35]. Blanching resulted in the leaching of the reducing sugars and amino acids into the solution, which decreased the Maillard reaction during frying, leading to a brighter color. Similarly, PEF can improve the permeability of cells and enable the leaching of reducing sugars and amino acids. However, certain reducing sugars and amino acids may remain on the surface of the samples, resulting in the color of the PEF-pretreated samples not being as bright as that of the blanched-pretreated samples; the color was darker at the edge of the samples. The total color change Δ*E* of the combined PEF + blanching-pretreatment was lower than that of the PEF-pretreated sample but higher than the blanched pretreated sample. This finding does not agree with the results reported by Zhang et al. [20], who found that the combined pretreatment of PEF + blanching significantly reduced the browning degree of French fries during frying. This may be because the trend in the total color change was not consistent for the various types of potatoes (regular potato and sweet potato).

(6) ∆E=a+bt

Here, *a* and *b* indicate the constants of the models.

### 3.4. Effect of Pretreatment on the Hardness of Sample

**Figure 6:**
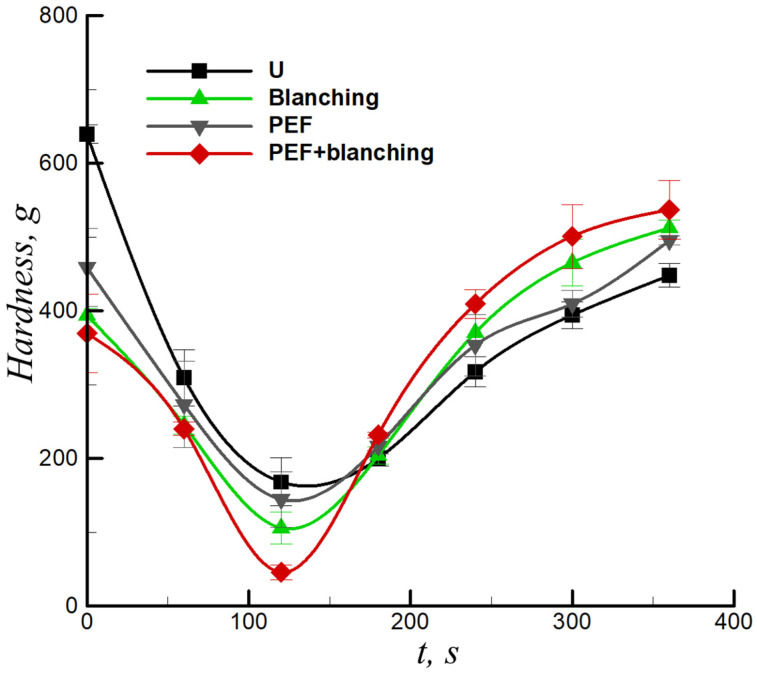
Hardness of sweet potato chips versus frying time for untreated (U), blanching-pretreated, PEF-pretreated, and combination of PEF + blanching-pretreated samples.

Textural characteristics during frying are indicators of the development of heat and mass transfer processes [36]. Figure 6 presents the hardness versus frying time for the untreated, blanching-pretreated, PEF-pretreated, and PEF + blanching-pretreated samples. Before frying, the hardness values of the pretreated samples (PEF, blanching, and PEF + blanching) were significantly lower than those of the untreated samples (Figure 6). The initial softening of tissues in the PEF-pretreated samples originated from an increase in the cell membrane permeability and cell breakdown [37]. A previous study reported that blanching pretreatment induces lamellar media solubilization and starch gelatinization as a result of tissue softening [38]. A similar softening effect on potato tissues following PEF + blanching was reported by Zhang et al. [20]. For all fried sweet potato chips (untreated and pretreated), the hardness values first decreased (*t* < 120 s) and then increased with frying time *t* > 120 s. This result agrees with previous studies regarding the textural properties of fried potatoes [15,29]. Note, the hardness of the blanching, PEF, and PEF + blanching-pretreated samples increased by 14.5%, 10.58%, and 19.92%, respectively, compared to those of the untreated samples at the end of frying (360 s). The final hardening of the samples reflects the formation of a surface crust during frying. Accordingly, the PEF and blanching pretreatment promoted water loss (Figure 3) in the sweet potato chips and reduced the adhesion between the cells, resulting in an increase in the hardness of the sweet potato chips after frying. Moreover, the formation of denser skin on the surface of the fried sample may restrict oil immersion while frying the sweet potato tissue (Figure 4).

### 3.5. Effect of Pretreatment on the Acrylamide Content of the Sample

**Figure 7:**
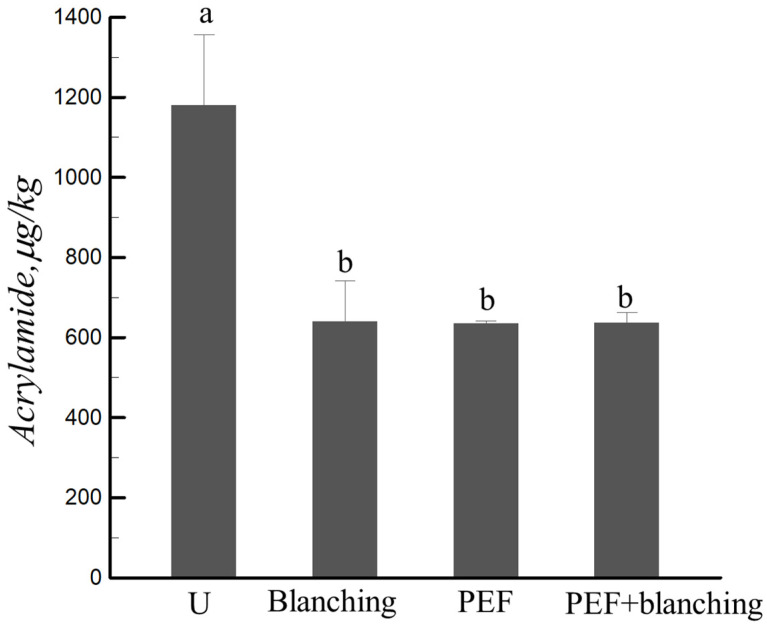
Effect of various pretreatments on the acrylamide content of fried sweet potato chips. ^a,b^ Values with different superscript letters means significantly difference.

In 2002, the Swedish National Food Administration found that carcinogen acrylamide (AA) was formed in heated starch-based foods [39]. Pedreschi et al. reported that toxic AA is a byproduct of the Maillard reaction of reducing sugars and amino acids during thermal processing [40]. The AA content in the fried sweet potato chips versus the pretreated chips is shown in Figure 7, which demonstrates that pretreatment (PEF, blanching, and PEF + blanching) significantly decreased the AA content in the fried samples by nearly 46.10% compared to the untreated samples. This can be explained by the increase in the frying rate with the pretreatment (less frying time, Figure 3); the leaching of reducing sugars and amino acids from the sweet potato slices by PEF and blanching can also decrease the acrylamide content during frying [41]. The Maillard reaction during frying is related to the degree of browning of the sample. The PEF + blanching pretreatment significantly decreased the color change of the sample (Figure 5) compared to the PEF-pretreated sample; however, there was no significant difference between the pretreatments for AA formation in frying sweet potato chips (Figure 7). Therefore, the formation of AA during frying is not only linked to color change but also to the reaction substrate, frying temperature, and frying time [42,43]. Liyanage et al. [44] demonstrated that an increase in temperature from 160 to 190 °C decreased the AA content by approximately 90%. They also found that the acrylamide formation in cultivars of Atlantic, Snowden, and Vigor pretreated by blanching in distilled water decreased by 19–59%. Genovese et al. reported that the AA reduction for potato chips pretreated by PEF (1.5 kV cm^−1^, 10 ms, 100 Hz) was 30%, whereas it was 17% for the hot water blanching pretreatment (85 °C, 3.5 min) [45].

### 3.6. Effect of Pretreatment on the Microstructure of a Sample

**Figure 8:**
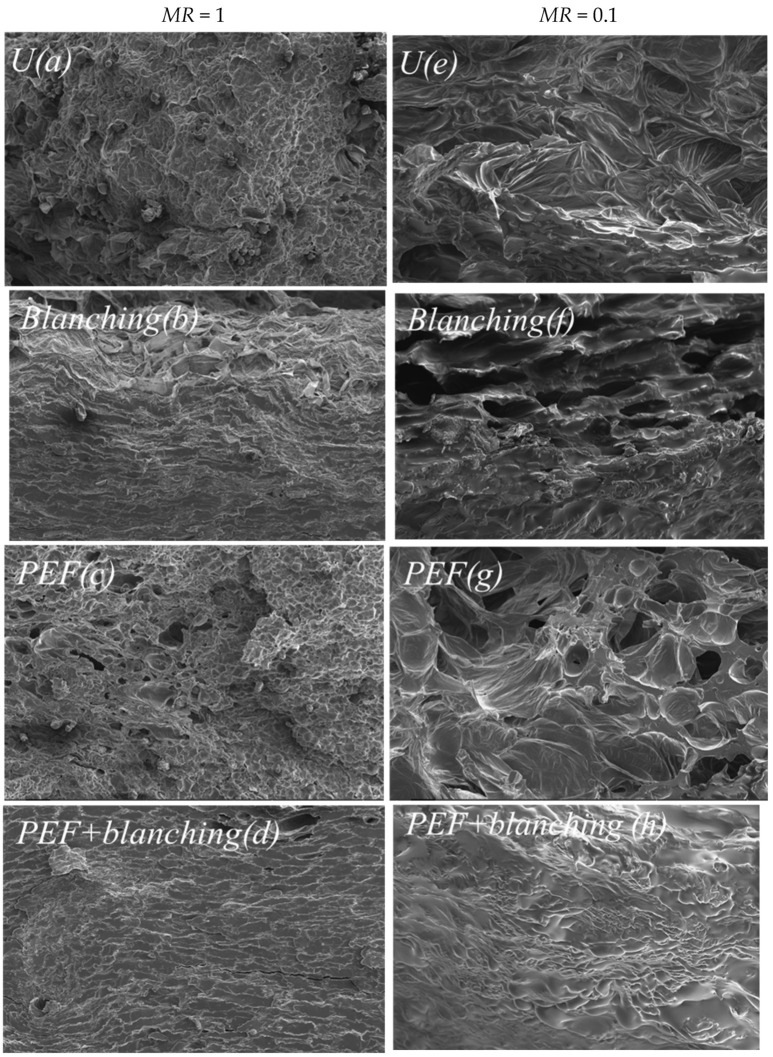
SEM images of fresh (*MR* = 1) (**a**) untreated(U), (**b**) blanching-pretreated, (**c**) PEF-pretreated, and (**d**) PEF + blanching-pretreated samples; and fried (*MR* = 0.1) (**e**) untreated(U), (**f**) blanching-pretreated, (**g**) PEF-pretreated, and (**h**) PEF + blanching-pretreated samples.

Figure 8 demonstrates the scanning electron micrographs of the cross-sections of fresh sweet potato slices (*MR* = 1, Figure 8a–d) and fried sweet potato chips (*MR* = 0.1 Figure 8e,f) for untreated and pretreated samples. Fresh sweet potato slices without pretreatment demonstrated largely intact and turgid tissues, and spherical starch granules were present in the parenchymal cellular compartments (Figure 8a). Nevertheless, the blanching pretreatment resulted in significant starch gelatinization and a smoother structure (Figure 8b). Due to electroporation, samples pretreated with PEF demonstrated pores on the cell wall of the sweet potato (Figure 8c), which is consistent with the results of the present studies [2,46]. As shown in Figure 8d, in samples pretreated by the combination of PEF + blanching, cracks were found on the sweet potato wall in addition to holes, which promotes the release of water from the surface at a higher rate during frying (Figure 3). After deep-frying, the cells did not exhibit a stereoscopic morphology, and the cell walls were disrupted and no longer upright, which is in agreement with the results of Zhang et al. [47]. In the untreated and pretreated fried samples, starch granules were no longer found and were swollen, gelatinized, and dehydrated during frying [46]. The PEF pretreatment increased the internal porosity (Figure 8g), which allowed the rapid evaporation of water during frying and prevented oil absorption. Furthermore, the internal structure of sweet potato chips obtained via the PEF + blanching pretreatment was smoother and flatter, thereby decreasing the oil absorption during frying processing (Figure 4).

## 4. Summary

Compared to the untreated samples, PEF, blanching, and PEF + blanching pretreatments reduced the final moisture ratio, oil content, acrylamide content, and total color change of the fried sweet potato chips. Furthermore, the combined PEF + blanching pretreatment significantly improved the quality of fried sweet chips when compared to the single or untreated methods. Note, the hardness of the blanching, PEF, and PEF + blanching-pretreated samples increased by 14.5%, 10.58%, and 19.92%, respectively, reflecting the formation of a surface crust during frying, and ultimately decreasing the oil content of the sample. Finally, chips pretreated by PEF + blanching had a lower oil (0.37 g/g DM) and acrylamide content (638 μg/kg). Cross-sectional SEM observations demonstrated that the internal structure of sweet potato chips obtained via the PEF + blanching pretreatment was smoother and flatter, thereby decreasing the oil absorption during frying. In conclusion, the combined PEF + blanching pretreatment is feasible for the future industrial production of frying root vegetables, which can finely control the deterioration of product quality and reduce operational and maintenance costs.

## Figures and Tables

**Table 1:** Parameters of the equation fitted for the oil content of untreated, blanching, PEF, and PEF + blanching-pretreated samples.

	a	b	R^2^
U	−0.23	−92.42	0.962
Blanching	−0.61	−126.63	0.956
PEF	−0.67	−76.73	0.956
PEF + blanching	−0.56	−99.71	0.902

**Table 2:** Parameters of the linear equation fitted for the total color change of untreated, blanched, PEF, and PEF + blanched pretreated samples.

	a	b	R^2^
U	12.96	0.027	0.977
Blanching	10.10	0.026	0.976
PEF	12.43	0.024	0.963
PEF + blanching	11.19	0.025	0.983

## Data Availability

The data presented in this study are available on request from the corresponding author.
